# Routine Vaccination Coverage — Worldwide, 2020

**DOI:** 10.15585/mmwr.mm7043a1

**Published:** 2021-10-29

**Authors:** Pierre Muhoza, M. Carolina Danovaro-Holliday, Mamadou S. Diallo, Padraic Murphy, Samir V. Sodha, Jennifer H. Requejo, Aaron S. Wallace

**Affiliations:** ^1^Epidemic Intelligence Service, CDC; ^2^Global Immunization Division, Center for Global Health, CDC; ^3^Department of Immunization, Vaccines and Biologicals, World Health Organization, Geneva, Switzerland; ^4^Division of Data, Analytics, Planning and Monitoring, UNICEF, New York, New York.

Endorsed by the World Health Assembly in 2020, the Immunization Agenda 2030 (IA2030) strives to reduce morbidity and mortality from vaccine-preventable diseases across the life course (*1*). This report, which updates a previous report (*2*), presents global, regional,* and national vaccination coverage estimates and trends as of 2020. Changes are described in vaccination coverage and the numbers of unvaccinated and undervaccinated children as measured by receipt of the first and third doses of diphtheria, tetanus, and pertussis-containing vaccine (DTP) in 2020, when the COVID-19 pandemic began, compared with 2019. Global estimates of coverage with the third dose of DTP (DTP3) and a polio vaccine (Pol3) decreased from 86% in 2019 to 83% in 2020. Similarly, coverage with the first dose of measles-containing vaccine (MCV1) dropped from 86% in 2019 to 84% in 2020. The last year that coverage estimates were at 2020 levels was 2009 for DTP3 and 2014 for both MCV1 and Pol3. Worldwide, 22.7 million children (17% of the target population) were not vaccinated with DTP3 in 2020 compared with 19.0 million (14%) in 2019. Children who did not receive the first DTP dose (DTP1) by age 12 months (zero-dose children) accounted for 95% of the increased number. Among those who did not receive DTP3 in 2020, approximately 17.1 million (75%) were zero-dose children. Global coverage decreased in 2020 compared with 2019 estimates for the completed series of *Haemophilus influenzae* type b (Hib), hepatitis B vaccine (HepB), human papillomavirus vaccine (HPV), and rubella-containing vaccine (RCV). Full recovery from COVID-19–associated disruptions will require targeted, context-specific strategies to identify and catch up zero-dose and undervaccinated children, introduce interventions to minimize missed vaccinations, monitor coverage, and respond to program setbacks (*3*).

In 1974, the World Health Organization (WHO) established the Expanded Programme on Immunization to ensure that all infants have access to four vaccines (Bacillus Calmette-Guérin vaccine [BCG], DTP, Pol, and MCV) to protect against six diseases (tuberculosis, diphtheria, tetanus, pertussis, poliomyelitis, and measles). Since then, additional vaccines and doses have been introduced during the first year of life (e.g., pneumococcal conjugate vaccine [PCV], rotavirus, RCV, HepB, and Hib) and later in childhood and adolescence (e.g., MCV2 and HPV) (*4*). WHO and UNICEF derive national vaccination coverage estimates through annual country-by-country review of available data, including administrative and survey-based coverage^†^^,^^§^ (*5*). DTP3 coverage by age 12 months is an indicator of immunization program performance. Children who do not receive DTP1 (zero-dose children) reflect a lack of access to immunization services. Those who receive DTP1 but do not complete the series are considered to have dropped out; they represent underutilization of immunization services among children with access.

WHO and UNICEF estimates during 2010–2019 indicate that global coverage with the DTP series stagnated, with coverage estimates ranging from 89% to 90% for DTP1 and from 84% to 86% for DTP3. From 2019 to 2020, global coverage declined from 90% to 87% for DTP1 and from 86% to 83% for DTP3, levels last observed in 2006 and 2009 for DTP1 and DTP3, respectively. In 2020, DTP1 coverage ranged from 79% in the WHO African Region to 97% in the European Region (Table 1). DTP3 coverage estimates ranged from 72% in the African Region to 95% in the Western Pacific Region. The Western Pacific Region was the only region with unchanged DTP3 coverage estimates from 2019 to 2020, whereas all others experienced decreases. Worldwide, the number of children who did not complete the 3-dose DTP series increased by 20% to 22.7 million from 2019 to 2020. Among them, 17.1 million (75%) were zero-dose children, and 5.6 million (25%) had started, but not completed, the DTP series. Approximately 95% of the increased number of children who failed to complete the DTP series between 2019 and 2020 (3.7 million) were zero-dose children. During 2019–2020, global DTP1-to-DTP3 dropout was stable at 4%–5%, ranging from 0.8% in the Western Pacific Region to 8% in the African Region.

**TABLE 1:** Vaccination coverage,* by vaccine and World Health Organization region — worldwide, 2020

Vaccine	No. (%) of countries with vaccine in schedule	WHO region % coverage^†^						
		Global	AFR	AMR	EMR	EUR	SEAR	WPR
BCG	156 (80)	85	79	68	89	94	87	95
DTP1	194 (100)	87	79	88	87	97	88	96
DTP3	194 (100)	83	72	82	81	94	85	95
HepB BD	114 (58)	42	6	60	35	41	51	84
HepB3	190 (98)	83	72	82	81	91	85	95
Hib3	192 (99)	70	72	81	81	79	83	25
HPV, last^§^	111 (57)	13	18	44	0	29	3	5
MCV1	194 (100)	84	68	85	83	94	88	95
MCV2	179 (92)	70	36	73	76	91	78	94
PCV3	148 (76)	49	68	76	52	79	27	16
Pol3	194 (100)	83	71	81	84	94	85	94
RCV1	173 (89)	70	36	85	45	94	87	95
Rota, last^¶^	114 (52)	46	53	71	53	30	58	2

**Abbreviations:** AFR = African Region; AMR = Region of the Americas; BCG = Bacille Calmette-Guérin vaccine; DTP3 = third dose of diphtheria and tetanus toxoids and pertussis-containing vaccine; EMR = Eastern Mediterranean Region; EUR = European Region; HepB BD = birth dose of hepatitis B vaccine; HepB3 = third dose of hepatitis B vaccine; Hib3 = third dose of *Haemophilus influenzae* type b vaccine; HPV, last = final dose of human papillomavirus vaccine; MCV1 = first dose of measles-containing vaccine; MCV2 = second dose of MCV; PCV3 = third dose of pneumococcal conjugate vaccine; Pol3 = third dose of polio vaccine; RCV1 = first dose of rubella-containing vaccine; Rota, last = final dose of rotavirus vaccine series; SEAR = South-East Asia Region; WHO = World Health Organization; WPR = Western Pacific Region.

* Summary tables of WHO recommendations for routine immunization. https://www.who.int/teams/immunization-vaccines-and-biologicals/policies/who-recommendations-for-routine-immunization---summary-tables

^†^ BCG coverage based on 156 countries with BCG in the national schedule; coverage for all other vaccines based on 194 countries (global) or all countries in the specified region. Administrative coverage is the number of vaccine doses administered to those in a specified target group divided by the estimated target population. During vaccination coverage surveys, a representative sample of households is visited and caregivers of children in a specified target group (e.g., those aged 12–23 months) are interviewed. Dates of vaccination are transcribed from the child's home-based record or from health facility records or are recorded based on caregiver recall. Survey-based vaccination coverage is calculated as the proportion of persons in a target age group who received a vaccine dose.

^§^ Number of doses to complete the HPV series depends on age of recipient.

^¶^ Number of doses to complete the rotavirus vaccine series varies among vaccine products.

The number of zero-dose children varied by WHO region, economic classification,^¶^ and country eligibility for support from Gavi, the Vaccine Alliance** (Table 2). During 2019–2020, the number of zero-dose children was stable in the European Region at 0.3 million but increased in the African (from 7.1 million to 7.7 million), Americas (from 1.6 million to 1.7 million), Eastern Mediterranean (from 1.8 million to 2.3 million), European (from 2.8 million to 3.4 million), South-East Asia (from 2.0 to 4.1 million), and Western Pacific (from 0.9 million to 1.0 million) regions (Figure). In 2020, middle-income countries had the largest number of zero-dose children (12.1 million; 71%); countries in the African and South-East Asia regions each accounted for 4.1 million (24%) children. Low-income countries accounted for 4.5 million (26%) zero-dose children. In 2020, 13.7 million (80%) zero-dose children lived in Gavi-eligible countries. Approximately two thirds (11.1 million; 65%) of zero-dose children in 2020 lived in 10 countries: Angola, Brazil, Democratic Republic of the Congo, Ethiopia, India, Indonesia, Mexico, Nigeria, Pakistan, and Philippines.

**TABLE 2:** Number and percentage of surviving infants not receiving the first dose of diphtheria and tetanus toxoids and pertussis-containing vaccine (zero-dose children), by World Health Organization region, Gavi eligibility, and World Bank economic classification — worldwide, 2010, 2019, and 2020

Characteristic/Year	WHO region*							Economic classification^†^			Among Gavi-eligible countries^§^
	Global^¶^	AFR	AMR	EMR	EUR	SEAR	WPR	Low	Middle	High	
**2010**											
Total no. of countries	193	46	35	21	53	11	27	35	106	49	67
No. of surviving infants (millions)	133.1	30.5	15	16.2	11.2	35.8	24.4	25.1	95.3	12.6	76.1
Global % of surviving infants	—	23	11	12	8	27	18	19	72	9	57
No. of zero-dose children (millions)	14.9	6.1	0.5	2.6	0.5	4.3	0.9	3.3	11.2	0.3	12.7
Global % of zero-dose children	—	41	3	17	3	29	6	22	75	2	85
**2019**											
Total no. of countries	194	47	35	21	53	11	27	29	103	60	67
No. of surviving infants (millions)	135.8	35.7	14.5	17.5	10.9	33.8	23.2	21.8	101.3	12.5	80.2
Global % of surviving infants	—	26	11	11	8	25	17	16	75	9	59
No. of zero-dose children (millions)	13.6	7.1	1.6	1.8	0.3	2.0	0.9	4.2	9.0	0.3	10.6
Global % of zero-dose children	—	52	12	13	2	15	6	31	66	2	78
**2020**											
Total no. of countries	194	47	35	21	53	11	27	27	108	57	68
No. of surviving infants (millions)	135.7	36.3	14.5	17.5	10.8	33.7	22.9	21.6	101.4	12.2	80.7
Global % of surviving infants	—	26	11	13	8	25	17	16	75	9	59
No. of zero-dose children (millions)	17.1	7.7	1.7	2.3	0.3	4.1	1.0	4.5	12.1	0.3	13.7
Global % of zero-dose children	—	45	10	13	2	24	6	26	71	2	80

**Abbreviations:** AFR = African Region; AMR = Region of the Americas; EMR = Eastern Mediterranean Region; EUR = European Region; GNI = gross national income; SEAR = South-East Asia Region; USD = U.S. dollars; WHO = World Health Organization; WPR = Western Pacific Region.

* Included countries are WHO member states (N = 193 for 2010; N = 194 for 2019 and 2020).

^†^ Low-income economies are defined as those with a GNI in USD per capita in 2010 of ≤$1,005, in 2019 of ≤$1,035, and in 2020 of ≤$1,045; middle-income economies are those with a GNI per capita in 2010 of $1,006–12,275, in 2019 of $1,036–12,535, and in 2020 of $1,046–12,695; high-income economies are those with a GNI per capita in 2010 of ≥$12,275, in 2019 of ≥$12,536, and in 2020 of ≥$12,696; calculated using the World Bank Atlas method (https://datahelpdesk.worldbank.org/knowledgebase/articles/906519-world-bank-country-and-lending-groups). Categorization is based on the World Bank’s economic classification for 2021. Cook Islands and Niue (WPR) are missing GNI data and are excluded from this categorization. Similarly, 2020 data for Venezuela were excluded as temporarily unclassified pending release of revised national accounts statistics.

^§ ^Based on Gavi 4.0 (2016–2020), eligibility includes 68 low- and middle-income countries eligible to receive financial assistance through grants contingent on a country’s GNI per capita. Eligibility is defined as a country’s average 3-year GNI per capita of ≤$1,580. As GNI increases, a country moves through Gavi’s different eligibility phases until reaching the transition phase when GNI exceeds the eligibility threshold. https://www.gavi.org

^¶^ Because of rounding, percentages across rows for regions do not necessarily sum to 100%.

**FIGURE:**
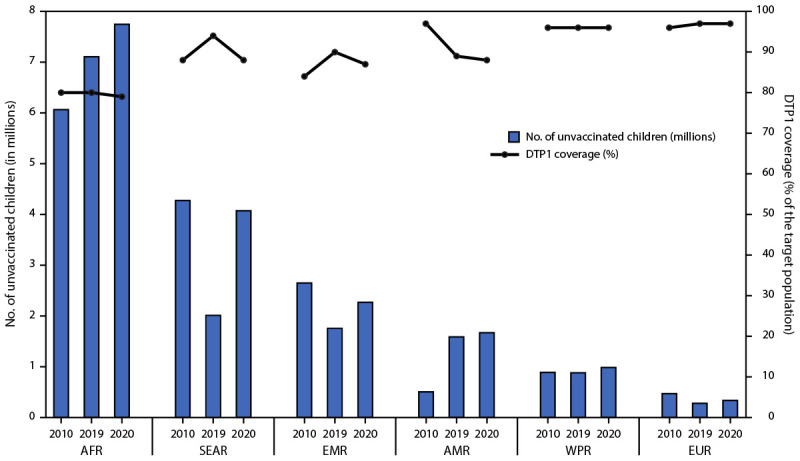
Estimated number of zero-dose children* during the first year of life and estimated coverage with first dose of diphtheria and tetanus toxoids and pertussis-containing vaccine, by World Health Organization region — worldwide, 2010, 2019, and 2020 **Abbreviations:** AFR = African Region; AMR = Region of the Americas; DTP1 = first dose of diphtheria and tetanus toxoids and pertussis-containing vaccine; EMR = Eastern Mediterranean Region; EUR = European Region; SEAR = South-East Asia Region; WPR = Western Pacific Region. * Zero-dose children are surviving infants who did not receive the first dose of DTP1 during the first year of life. Increase in the number of zero-dose children in the African Region reflects population growth.

During 2010–2019, global coverage with MCV1 stagnated between 84% and 86%, while MCV2 coverage increased from 42% to 71%, reflecting second dose introductions in many countries.^††^ From 2019 to 2020, global MCV1 coverage decreased to the 2014 level of 84%, whereas MCV2 coverage was relatively stable at 71% in 2019 and 70% in 2020. MCV1 coverage in 2020 ranged from 68% in the African Region to 95% in the Western Pacific Region (Table 1). Among all countries, MCV2 coverage varied from 36% in the African Region to 94% in the Western Pacific Region.

During 2019–2020, global coverage decreased for the completed series of Hib vaccine (from 72% to 70%), RCV vaccine (from 71% to 70%), HepB (3-dose series: from 85% to 83%; birth dose stable at 42%), and HPV (from 15% to 13%). Global coverage with the completed PCV series remained stable at 49%, whereas rotavirus vaccination coverage increased from 39% to 46%. One country introduced PCV, and seven countries introduced rotavirus vaccine (Table 1).

## Discussion

Following high (although stagnant) routine vaccination coverage during 2010–2019, a notable decline in global coverage for most vaccines occurred from 2019 to 2020. Although this decrease represented only a few percentage points, approximately 3 million more children did not complete the infant vaccination series in 2020. Even vaccines with apparent stable or increased coverage (i.e., MCV2, PCV, and rotavirus) were adversely affected. However, the drops in global coverage were offset by recent vaccine or dose introductions in some countries. The decrease in coverage in 2020 is likely related to effects of the COVID-19 pandemic. Surveys conducted in 2020 to gauge immunization program disruptions indicated decreased access because of physical distancing and transportation reductions, concerns by caregivers and health workers about COVID-19 exposure, and supply chain interruptions (*6*). The impacts to immunization coverage in 2020 were variable across regions and countries, with the South-East Asia and Eastern Mediterranean regions experiencing the largest declines in DTP3 coverage. DTP3 coverage in the Americas has continued a downward trend since 2016 (*2*).

Zero-dose children tend to live in vulnerable communities served by outreach services that are more prone to disruption and less resilient to recovery (*6*). Extending immunization services to reach zero-dose children and communities is one of the objectives of IA2030 and the Gavi 5.0 strategy (*1*,*7*). Achieving this objective requires an understanding of the socioeconomic, cultural, geographic, and systemic barriers to vaccination in these communities and the development of appropriate, context-specific strategies to increase access, availability, and demand for immunization services (*8*).

Although evidence suggests that routine immunization began to recover toward the end of 2020 (*6*), catch-up vaccination strategies and continued monitoring are essential to address the immunity gaps caused by immunization program disruptions (*9*,*10*). Catch-up strategies might include more immediate activities, such as mass vaccination activities and targeted communication to persons identified as having missed vaccine doses. Countries should also develop a catch-up vaccination framework within routine immunization, which could include modifying immunization policies, improving defaulter tracking, training health workers to incorporate catch-up strategies into the immunization program, screening children for vaccination status at any health service encounter or at school entry, and expanding age-based eligibility for vaccinations to ensure that unvaccinated older children receive missed vaccines. A robust catch-up framework could also strengthen program resilience to withstand large-scale disruptions because the program could, at lower cost, rely on the routine immunization program to identify and administer missed vaccines to children rather than depending solely on costly mass vaccination events.

The findings in this report are subject to at least five limitations. First, 2019 data were used for 35 countries that did not report 2020 data; however, these countries included <5% of the 2020 global birth cohort.^§§^ Second, data quality limitations could have resulted in inaccurate estimations of administrative coverage (*5*). Third, sampling and recall bias could have affected survey-based estimates of coverage (*5*). Fourth, estimates for 2020 are not directly informed by survey data in all countries because of survey implementation disruptions. Finally, estimates do not include statistical uncertainty.

Action is urgently needed to address immunity gaps caused by pandemic-related disruptions in immunization delivery to prevent vaccine-preventable disease outbreaks in countries with health systems already burdened by COVID-19. Reversing worrisome trends in some countries and extending previous gains in vaccination coverage beyond prepandemic levels will require targeted and context-specific approaches to eliminate barriers to vaccination, particularly in communities with large populations of zero-dose children. Defining country-specific strategies to identify missed children, minimize missed opportunities for vaccination, and implement catch-up vaccination is critical to lessen the impact of the COVID-19 pandemic on progress toward achieving global immunization goals.

**Summary**

**What is already known about this topic?**

Global coverage with the third dose of diphtheria and tetanus toxoids and pertussis-containing vaccine (DTP3) and of polio vaccine (Pol3) and the first dose of measles-containing vaccine (MCV1) remained between 84% and 86% during 2010–2019.

**What is added by this report?**

In 2020, estimated global coverage with DTP3 and Pol3 decreased to 83%; MCV1 coverage decreased to 84%. Globally, 17.1 million zero-dose children did not receive the first DTP dose, an increase of 3.5 million from 2019.

**What are the implications for public health practice?**

Full recovery from COVID-19–associated disruptions will require targeted, context-specific strategies to identify and catch up zero-dose and undervaccinated children, introduce interventions to minimize missed vaccinations, monitor coverage, and respond to program setbacks.

## Footnotes

* Based on World Health Organization regional classifications. https://www.who.int/about/who-we-are/regional-offices

† For a given vaccine, the administrative coverage is the number of vaccine doses administered to persons in a specified target group divided by the estimated target population. Doses administered during routine immunization visits are counted, but doses administered during supplemental immunization activities (mass campaigns) usually are not. During vaccination coverage surveys, a representative sample of households is visited, and caregivers of children in a specified target age group (e.g., 12–23 months) are interviewed. Dates of vaccination are transcribed from the child’s home-based record, recorded based on caregiver recall, or transcribed from health facility records. Survey-based vaccination coverage is calculated as the proportion of persons in a target age group who received a vaccine dose.

§ For 35 countries that did not report immunization coverage data for 2020 by July 6, 2021, estimated coverage in 2019 was used. These countries represent <5% of the global birth cohort in 2020. Among the 35 countries, 17 were from the European Region, seven were from the Western Pacific Region, and five were from the African Region. WHO/UNICEF estimates of national immunization coverage are available at https://www.who.int/teams/immunization-vaccines-and-biologicals/immunization-analysis-and-insights/global-monitoring/immunization-coverage/who-unicef-estimates-of-national-immunization-coverage.

¶ Low-income economies are defined as those with a gross national income (GNI) in USD per capita in 2010 of ≤$1,005, in 2019 of ≤$1,035 and in 2020 of ≤$1,045; middle-income economies are those with a GNI per capita in 2010 of $1,006–12,275, in 2019 of $1,036–$12,535 and in 2020 of $1,046–$12,695; high-income economies are those with a GNI per capita in 2010 of ≥$12,275, in 2019 of ≥$12,536, and in 2020 of ≥$12,696; calculated using the World Bank Atlas method (https://datahelpdesk.worldbank.org/knowledgebase/articles/906519-world-bank-country-and-lending-groups). Cook Islands and Niue (Western Pacific Region) are missing GNI data and are excluded from this categorization.

** Gavi, the Vaccine Alliance, is a public-private global health partnership with the goal of increasing access to immunization in poor countries. Country eligibility is defined by an average 3-year GNI per capita of ≤$1,580. Based on Gavi 4.0 (2016–2020), eligibility includes 68 low- and middle-income countries eligible to receive financial assistance through grants contingent on a country’s GNI per capita. As GNI increases, a country moves through Gavi’s different eligibility phases until reaching the transition phase, when GNI exceeds the eligibility threshold. https://www.gavi.org

†† During 2010–2019, 42 countries introduced the second dose of MCV2 into their immunization schedule. In 2020, only Madagascar introduced MCV2 into its immunization schedule.

§§ Given that these countries represent <5% of the global birth cohort in 2020, the missing data likely had a limited impact on reported estimates.
