# *Erratum:* Vol. 70, No. 43

**DOI:** 10.15585/mmwr.mm7046a7

**Published:** 2021-11-19

In the report “Routine Vaccination Coverage — Worldwide, 2020,” on page 1497, in the first column, the first full sentence should have read, “During 2019–2020, the number of zero-dose children was stable in the European Region at 0.3 million but increased in the African (from 7.1 million to 7.7 million), Americas (from 1.6 million to 1.7 million), Eastern Mediterranean (from 1.8 million to 2.3 **million), South**-East Asia (from 2.0 to 4.1 million), and Western Pacific (from 0.9 million to 1.0 million) regions (Figure).”

